# Improvement of quality of life and symptom burden after robot-assisted radical prostatectomy in patients with moderate to severe LUTS

**DOI:** 10.1038/s41598-021-95525-2

**Published:** 2021-08-18

**Authors:** Sami-Ramzi Leyh-Bannurah, Christian Wagner, Andreas Schuette, Nikolaos Liakos, Theodoros Karagiotis, Mikolaj Mendrek, Pawel Rachubinski, Katarina Urbanova, Matthias Oelke, Jorn H. Witt

**Affiliations:** Prostate Center Northwest, Department of Urology, Pediatric Urology and Uro-Oncology, St. Antonius-Hospital, Gronau, Germany

**Keywords:** Prostate, Prostatic diseases, Urinary tract obstruction

## Abstract

The aim of this study was to assess clinically meaningful differences of preoperative lower urinary tract symptoms (LUTS) and quality of life (QoL) before and after robot-assisted radical prostatectomy (RARP). Therefore we identified 5506 RARP patients from 2007 to 2018 with completed International Prostate Symptom Score (IPSS) and -QoL questionnaires before and 12 months after RARP in our institution. Marked clinically important difference (MCID) was defined by using the strictest IPSS-difference of − 8 points. Multivariable logistic regression analyses (LRM) aimed to predict ∆IPSS ≤ − 8 and were restricted to RARP patients with preoperatively moderate (IPSS 8–19) vs. severe (IPSS 20–35) LUTS burden (n = 2305). Preoperative LUTS was categorized as moderate and severe in 37% (n = 2014) and 5.3% of the complete cohort (n = 291), respectively. Here, a postoperative ∆IPSS ≤ − 8, was reported in 38% vs. 90%. In LRM, younger age (OR 0.98, 95%CI 0.97–0.99; p = 0.007), lower BMI (OR 0.94, 95%CI 0.92–0.97; p < 0.001), higher preoperative LUTS burden (severe vs. moderate [REF.] OR 15.6, 95%CI 10.4–23.4; p < 0.001), greater prostate specimen weight (per 10 g, OR 1.12, 95%CI 1.07–1.16; p < 0.001) and the event of urinary continence recovery (OR 1.66 95%CI 1.25–2.21; p < 0.001) were independent predictors of a marked LUTS improvement after RARP. Less rigorous IPSS-difference of − 5 points yielded identical predictors. To sum up, in substantial proportions of patients with preoperative moderate or severe LUTS a marked improvement of LUTS and QoL can be expected at 12 months after RARP. LRM revealed greatest benefit in those patients with preoperatively greatest LUTS burden, prostate enlargement, lower BMI, younger age and the event of urinary continence recovery.

## Introduction

Prostate cancer (PCa) and benign prostatic hyperplasia (BPH) occur in men of advanced age and are frequently coexistent. Both may be associated with lower urinary tract symptoms (LUTS) and increased serum PSA levels. To date, it remains unclear whether the association of PCa and BPH reflects a causal link, shared risk factors or pathophysiological mechanisms or detection bias^1^. Specifically, men suffering from LUTS often seek medical advice and receive PSA testing as part of the clinical evaluation^2–5^. In consequence, a substantial proportion of patients diagnosed with PCa and scheduled for radical prostatectomy (RP) suffer from preoperatively clinically important LUTS^3–5^. Such LUTS usually reduce patients’ quality of life (QoL), driven by either storage- (e.g. urgency, nocturia) or voiding-symptoms (e.g. intermittency, weak voiding stream)^6,7^ and can even have an impact on mortality risk^8^. Due to such impact on QoL, conventional oncological and functional outcomes, which are integral for PCa patient counselling, should be complemented with the postinterventional prospect of changed symptom burden and quality of life especially in the patients suffering from moderate or severe LUTS, which is often overlooked^9^.

For outcomes after RARP, this neglect is reflected in the fact that most series focus on postoperative urinary continence recovery^10,11^, whereas corresponding data on LUTS and QoL is rather sparse^12^. Moreover, most published series, which focus on latter outcome, are limited by small sample sizes^10,11,13,14^, particularly of RARP patients with moderate or severe preoperative LUTS^2,10^. Accordingly, most series rely on exploratory analyses, but forego multivariable analyses (MVA)^10,15,16^. Similarly, most series focus on changes of mean IPSS or QoL scores but do so without reporting minimal or marked clinically important differences (MCIDs) of pre- vs. postoperative LUTS. Such MCIDs allow easier clinically meaningful interpretation. Finally, aforementioned RARP studies rarely differentiate between storage (e.g. frequency, urgency, nocturia) and voiding-symptoms (e.g. intermittency, weak voiding stream)^17^. Former might indicate urge incontinence or underlying medical conditions. In consequence, thorough preoperative IPSS evaluation may be essential to better estimate chances of postoperative continence recovery, too^2,11^.

Thus, we assessed clinically meaningful marked and moderate differences of pre- vs. postoperative LUTS and associated QoL 12 months after RARP and identified predictors of post-RARP improvement.

## Material and methods

Within our database, we identified 11,711 PCa patients, who were treated with RARP in our hospital between 5/2006 and 12/2018. Inclusion criteria consisted of complete information on preoperative PSA and pathological characteristics. Functional parameters included urinary function and International Prostate Symptom Score (IPSS) and -QoL questionnaire-derived lower urinary tract symptoms (LUTS) burden preoperatively and at 12 months follow-up. All patients were preoperatively continent. Patients with radiation therapy of the pelvis or prostate (neoadjuvant/salvage/adjuvant), or local therapy of the prostate or bladder, or suspected metastases at preoperative staging were excluded. Finally, we selected 5506 RARP patients for further analysis. Preoperative medication such as: alpha-blocker therapy, 5-alpha-reductase inhibitor therapy, neoadjuvant antiandrogen therapy and/or luteinizing hormone-releasing hormone antagonist or agonist therapy (henceforth referred to as neoadjuvant ADT) did not represent exclusion criteria. No patients received adjuvant or salvage radiation therapy within that timeframe according to our exclusion criteria. The institutional review board at the St. Antonius-Hospital, Gronau, approved the retrospective study design and access to the patients’ medical records. All methods were carried out in accordance with the Declaration of Helsinki. Written informed consent was obtained from individual participants in the study.

### Outcomes

Urinary continence recovery was defined as a combination of 1) a score ≤ 2 for both the first and second question of the International Consultation on Incontinence Questionnaire-Urinary Incontinence Short Form (Q1 “*How often do you leak urine*?” and Q2 “*How much urine do you usually leak (whether you wear protection or not)?*”)^18^, 2) International Continence Society male questionnaire score ≤ 1 for the questions 2, 3, 4 and finally up to one pad usage within 24 h. Preoperatively, no pad usage was permitted for being deemed continent.

The IPSS score ranges from 0 to 35 points. It consists of the sum of 7 questions, each with a point range from 0 to 5, and one additional QoL question (“If you were to spend the rest of your life with your urinary condition just the way it is now, how would you feel about that?”, ranging from 0 (delighted), through 3 (mixed), to 6 (terrible). Symptom burden is categorized as “nothing or mild” (range 0–7 points), “moderate” (range 8–19) or “severe” (range 20–35). The IPSS voiding-subdomain consists of the questions 1, 3, 5 and 6 and ranges from 0 to 20. The IPSS storage-subdomain consists of the questions 2, 4 and 7 and ranges from 0 to 15. Finally, ≤ 2, 3 and > 3 QoL points were categorized as satisfied, mixed and unsatisfied QoL, respectively.

For 12 months after RARP we used the strictest criterion of IPSS-difference of at least 8 points (∆IPSS 8) in relation to the preoperative IPSS score^19–21^, this was defined as a marked clinical important difference (MCID). Specifically, a ∆IPSS ≤ − 8 represents a marked improvement, whereas a ∆IPSS ≥  + 8 would represent a marked deterioration. It is important to note that a marked improvement ∆IPSS ≤ − 8 cannot be observed in those patients with lowest symptom burden (range 0–7 points), since a ∆IPSS ≤ − 8 exceeds any value within that point range. A less rigorous, moderate clinical important difference of ∆IPSS 5 was also utilized^15,19–21^.

### Statistical analyses

Proportions of the MCIDs ∆IPSS 8 and 5 were calculated and stratified according to preoperative LUTS burden mild, moderate, and severe. T-Test was used to compare mean change of IPSS and QoL score per MCID.

Multivariable logistic regression analyses (LRM) aimed to predict a marked improvement, i.e. MCID ∆IPSS ≤ − 8 after 12 months within those with moderate and severe preoperative LUTS.

LRM were adjusted for age, PSA, preoperative LUTS burden (moderate [REF.] vs. severe), prostate specimen weight (g), the Postsurgical Cancer of the Prostate Risk Assessment Score (CAPRA-S)^22^, nerve-sparing (none vs. uni- vs. bilateral) and bladder neck reconstruction width (1–5 cm). The CAPRA-S is calculated with pre-surgical PSA, pathological Gleason score, surgical margin status, extracapsular extension, seminal vesicle invasion and lymph node invasion^22^. Similarly, as supplemental analyses, we also performed multivariable linear regression analyses for change of LUTS 12 months after RARP within the total cohort, i.e. those with any preoperative LUTS burden (IPSS range 0–35). Adjustment variables were the same as in LRM.

## Results

### Demographics

Within the total cohort, median values of age, BMI, PSA and prostate weight were 65 yrs. (IQR 60–70), 26.5 kg/m^2^ (IQR 24.6–28.7), 7.3 ng/ml (IQR 5.3–10.6) and 45 g (IQR 36–58), respectively (Table 1). Of all 5506 patients 58% had no or mild LUTS, whereas 37% and 5.3% had a moderate to severe LUTS, respectively. Patients with moderate and severe LUTS had lower rates of bilateral NS compared to the patients with no or mild LUTS, 84 and 78% vs. 86%, respectively (p < 0.001). Median surgical bladder neck reconstruction width was constant in all three groups, 2 cm (IQR 2–3). Based on CAPRA-S, 53, 32 and 15% of the patients were categorized as low-, medium and high-risk.

**Table 1:** Baseline characteristics of 5506 prostate cancer patients treated with robot-assisted radical prostatectomy at the Prostate Cancer Center Northwest, Gronau, Germany between 2006 and 2018 stratified according to the preoperative LUTS.

Value	All patients (n = 5506)		No or mild LUTS (n = 3201)		Moderate LUTS (n = 2014)		Severe LUTS (n = 291)		*p*-value
**Age, years, median (IQR)**	65	(60–70)	64	(59–69)	66	(61–71)	66	(61–71)	≥ 0.3^a^
**BMI, kg/m2, median (IQR)**	26.5	(24.6–28.7)	26.5	(24.7–28.7)	26.5	(24.5–28.7)	26.8	(24.5–29.1)	≥ 0.6^a^
**Preoperative PSA, ng/ml, median (IQR)**	7.3	(5.3–10.6)	7.2	(5.3–10.5)	7.3	(5.3–10.9)	7.3	(5.3–10.1)	≥ 0.2^a^
**Prostate weight, g, median (IQR)**	45	(36–58)	42	(34–53)	49	(39–64)	53	(42–67)	≥ 0.2^a^
**Charlson Comorbidity Index (CCI), n (%)**									
0	3243	59%	1945	61%	1142	57%	156	54%	0.003
1	1285	23%	731	23%	480	24%	74	25%	
2	626	11%	341	11%	254	13%	31	11%	
≥ 3	352	6.4%	184	5.8%	138	6.9%	30	10%	
**Preoperative IPSS score, median (IQR)**	6	(3–11)	4	(2–5)	11	(9–14)	23	(21–25)	≥ 0.3^a^
**Preoperative QoL score, median (IQR)**	1	(1–2)	1	(0–1)	2	(1–3)	4	(3–5)	0.04^b^
									≥ 0.7^a^
**Preoperative alpha-blocker-therapy, n (%)**	68	1.2%	21	0.7%	30	1.5%	17	5.8%	< 0.001
**Preoperative 5-alpha-reductase inhibitor therapy, n (%)**	119	2.2%	47	1.5%	57	2.8%	15	5.2%	< 0.001
**Neoadjuvant androgen deprivation therapy, n (%)**	289	5.3%	142	4.4%	123	6.1%	24	8.3%	0.002
**Surgical expertise, number of cases, median (IQR)**	799	(190–2397)	786	(184–2440)	815	(201–2374)	742	(187–2007)	0.4^a^
**Bladder neck reconstruction width, cm, median (IQR)**	2	(2–3)	2	(2–3)	2	(2–3)	2	(2–3)	≥ 0.2^a^
**Nerve sparing procedure, n (%)**									
None	193	3.5%	89	2.8%	78	3.9%	26	8.9%	< 0.001
Unilateral	651	11.8%	373	11.7%	239	11.9%	39	13.4%	
Bilateral	4662	84.7%	2739	85.6%	1697	84.3%	226	77.7%	
**Pathological ISUP grade, n (%)**									
1	1784	32%	988	31%	691	34%	105	36%	< 0.001
2	1715	31%	1055	33%	577	29%	83	29%	
3	1218	22%	742	23%	427	21%	49	17%	
≥ 4	789	14%	416	13%	319	16%	54	19%	
**Pathological tumor stage, n (%)**									
pT2	3646	66%	2117	66%	1337	0.6639	192	66%	< 0.001
pT3a	1323	24%	816	25%	451	22%	56	19%	
≥ pT3b	537	10%	268	8.4%	226	11%	43	15%	
**Lymph node invasion, n (%)**	402	7.3%	190	5.9%	182	9.0%	30	10.3%	< 0.001
**CAPRA-S risk group, n (%)**									
Low risk	2910	53%	1688	53%	1057	52%	165	57%	< 0.001
Intermediate risk	1774	32%	1074	34%	630	31%	70	24%	
High risk	822	15%	439	14%	327	16%	56	19%	

CAPRA-S: the Postsurgical Cancer of the Prostate Risk Assessment Score; IPSS: International Prostate Symptom Score; ISUP: International Society of Urological Pathology; LUTS: lower urinary tract symptoms; QoL: quality of life.

^a^Denotes insignificant p-values for each comparison between the LUTS severity categories, none or mild vs. moderate LUTS, none or mild vs. severe LUTS and moderate vs. severe LUTS. ^b^Comparison no or mild vs. moderate LUTS.

### Follow-up and outcomes

Median follow-up of patients without urinary continence recovery was 24 months (IQR 13–49). At 12 months post-RARP, urinary continence recovery rate was 90%. Preoperative vs. 12 months post-RARP median IPSS and QoL scores decreased from 6 (IQR 3–11) to 1 (IQR 1–2) and from 4 (IQR 2–6) to 1 (IQR 0–2), respectively. Similarly, median IPSS voiding-subdomain decreased from 3 (IQR 1–6) to 1 (IQR 0–3), whereas median IPSS storage-subdomain remained virtually unchanged, from 3 (IQR 2–5) to 3 (IQR 2–4). These mean changes of IPSS scores corresponded to preoperative vs. 12 months post-RARP proportions of ≤ mild, moderate and severe LUTS of 58 vs. 82%, 37 vs. 17% and 5.3 vs. 1.1%, respectively. Similarly, satisfied, mixed and unsatisfied QoL proportions were 77 vs. 84%, 13 vs. 9.3% and 10 vs. 6.8%, respectively.

A marked improvement, ∆IPSS ≤ − 8, translated to a QoL mean change of  − 1.66 (vs. + 0.14; p < 0.001). Conversely, a marked deterioration, ∆IPSS ≥  + 8 translated to a QoL mean change of + 2.26 (vs. − 0.27; p < 0.001).

**Figure 1:**
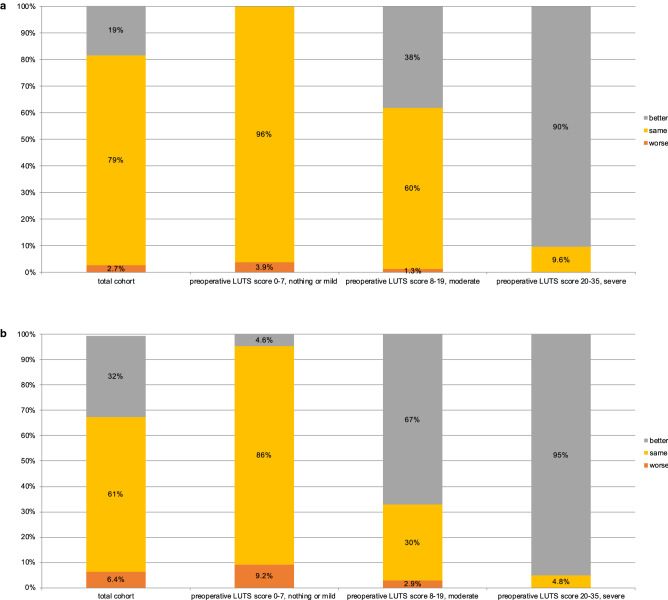
(**a**) "Marked clinically important differences 12 months after robot-assisted radical prostatectomy, defined as a change of at least 8 points in the International Prostate Symptom Score, in the overall cohort and stratified according to preoperative lower urinary tract symptoms burden, nothing or mild vs. moderate vs. severe". (**b**) "Moderate clinically important differences 12 months after robot-assisted radical prostatectomy, defined as a change of at least 5 points in the International Prostate Symptom Score, in the overall cohort and stratified according to preoperative lower urinary tract symptoms burden, nothing or mild vs. moderate vs. severe".

Stratification according to preoperative LUTS revealed great differences applying a MCID ∆IPSS 8 (Fig. 1a): Within patients with mild baseline LUTS (n = 3201), 3.9% showed a marked deterioration and 96% no change. Within patients with moderate baseline LUTS (n = 2014), 1.3% showed a marked deterioration, 60% no change and 38% experienced a marked improvement. Finally, within those with severe baseline LUTS (n = 291), 9.6% had no change and 90% had a marked improvement. Patterns were similar, but less distinct for different MCIDs ∆IPSS 5 (Fig. 1b).

### LRM analysis of predictors of marked clinical important difference (MCID)

LRM in those RARP patients with moderate or severe preoperative LUTS revealed younger age (OR 0.98, 95% CI 0.97–0.99; p = 0.007), lower BMI (OR 0.94, 95%CI 0.92–0.97; p < 0.001), higher preoperative LUTS burden (severe vs. moderate [REF.] OR 15.6, 95%CI 10.4–23.4; p < 0.001) and greater prostate specimen weight (per 10 g, OR 1.12, 95%CI 1.07–1.16; p < 0.001) as independent predictors of a marked improvement 12 months after RARP, ∆IPSS ≤ − 8 (i.e. at least 8 points less at 12 months after RARP; Table 2). Moreover, urinary continence recovery within 12 months was also a predictive factor for a marked improvement (OR 1.66 95%CI 1.25–2.21; p < 0.001). LRM results were virtually identical for moderate MCIDs ∆IPSS 5 (Table 2). Multivariable linear regression analyses confirmed same predictors within the total cohort with exception of age (Supplemental Table 1).

**Table 2:** Multivariable logistic regression model for prediction of marked and moderate LUTS improvement after robot-assisted radical prostatectomy in patients with moderate or severe preoperative LUTS.

Value	Marked LUTS improvement ∆IPSS ≤ − 8			Moderate LUTS improvement ∆IPSS − 5		
	OR^a^	95% CI	*p*-value	OR^a^	95% CI	*p*-value
Age, years, cont.	0.98	0.97–0.99	0.007	0.98	0.96–0.99	0.003
BMI, kg/m^2^, cont.	0.94	0.92–0.97	< 0.001	0.96	0.94–0.99	0.003
Preoperative LUTS burden (severe vs. moderate [REF.])	15.6	10.4–23.4	< 0.001	9.7	5.6–16.9	< 0.001
Prostate gland weight, cont. (per 10 g)	1.12	1.07–1.16	< 0.001	1.18	1.12–1.24	< 0.001
Urinary continence recovery within 12 months after RARP, cont.	1.66	1.25–2.21	< 0.001	1.84	1.40–2.40	< 0.001

CAPRA-S: the postsurgical Cancer of the Prostate Risk Assessment score; IPSS: International Prostate Symptom Score; LUTS: lower urinary tract symptoms; RARP: robot-assisted radical prostatectomy.

^a^Adjusted for surgical experience, preoperative PSA, preoperative medication with alpha-blockers, 5-alpha-reductase inhibitors, neoadjuvant androgen deprivation, CAPRA-S score, nerve-sparing status and bladder neck reconstruction width.

## Discussion

Previous series with multivariable analyses on examining postoperative changes of LUTS were sparse, heterogeneous and limited by specific selection of variables or small sample size. Thus, we aimed to provide most comprehensive analyses of marked and moderate clinically important differences between pre- vs. post-RARP LUTS and associated QoL in a large cohort of PCa patients. Our findings aim to provide robust decision factors if local PCa treatment such as RARP should be considered as primary therapy^9^.

Our study revealed several important findings. First, there was a substantial proportion of 42% of PCa patients with moderate or severe preoperative LUTS (n = 2305). These patient numbers are more than 30-fold compared to previous series^2,10^. Such sample size represents a prerequisite to identify predictors of LUTS improvement in multivariable analyses. Moreover, such extent of pre-existing LUTS burden and associated negative impact on QoL might greatly confound the perceived outcome after local treatment such as RARP, despite otherwise favourable functional and oncological outcomes. In consequence, it is of utmost importance to consider the treatment of the bothering LUTS as core part of the overall therapy^23^.

Second, RARP patients with moderate and severe preoperative LUTS showed substantial marked clinical improvement rates of 60 and 90%, respectively. It is of note that within the remaining 40% of former group with moderate LUTS, less than 2% experience a marked deterioration. Thus, we display great safety of RARP in this regard, at least in experienced centers. Conversely, we not only confirm postoperative improvement, but even demonstrate greater potential of LUTS improvement after RARP, even beyond previous reports^15^, since we applied the most rigorous MCID ∆IPSS ≤ − 8.

Third, there is a statistically significant association between LUTS burden and unfavourable pathological cancer characteristics as evidenced by increasing proportions of CAPRA-S high-risk from 14 to 19% (p < 0.001). These findings support the proposed link between BPH and increased risk of PCa and PCa-related mortality from epidemiological studies^1,24^. Since inverse stage migration trends demonstrate increasing proportions of senior PCa patients with unfavourable PCa characteristics, who receive local treatment such as RARP, those simultaneously suffering from moderate or severe LUTS will be even more prevalent^25,26^.

Fourth, we clearly demonstrated that the RARP-mediated effect on postoperative LUTS is driven by voiding symptoms. This is in line with a previously proposed substratification^2,27^ and series that compared pre- vs. postoperative voiding metrics^28^. In consequence, during surgical work-up the overall IPPS score as well as the voiding-subdomain should be evaluated. Specifically, a larger score in the voiding-subdomain indicates greater possible RARP-mediated improvement effect on LUTS.

Fifth, the study at hand is one of the few to provide LRM to predict marked post-RARP improvement of LUTS^2,10,15,16,27^. We confirm preoperative LUTS burden as a strong predictor^14–16^. Moreover, larger prostate size and younger age were predictors as well, which are conceivable due to shared risk factors between BPH, associated LUTS and PCa^1,24^. Specifically, with regard to age, a longitudinal study impressively demonstrated LUTS progression in men over a period of 11 years^29^.

We also identified lower BMI as predictor. Several series demonstrated LUTS exacerbation in obese men^2^, often even unrelated to PCa^30–33^. In consequence, for those with residual post-RARP LUTS, obesity remains an important target for intervention, which can be already addressed while counselling the patient.

Finally, not surprisingly, urinary continence recovery is contributive for a marked improvement. It is of note that urinary continence recovery is neither a patient characteristic nor a preoperative condition unlike the aforementioned predictors, but a key functional outcome after RARP. Our findings confirm such association between this outcome and LUTS burden^2,27^. Interestingly, to our knowledge, we are the first to show these predictors in such multivariable fashion. For example, the Japanese RARP series by Haga et al.^14^ confirmed our finding of preoperative LUTS burden as a main predictor. However, they identified nerve-sparing as predictor, which we are not able to confirm, potentially due to over 96% of patients who had at least unilateral nerve-sparing in our cohort. Conversely, they neither confirmed BMI, nor age nor prostate weight. As acknowledged by the authors, the sample size (n = 200) was limited. Moreover, they did not utilize MCIDs and their proportion of those with moderate or severe LUTS was not categorized. Finally, we included neoadjuvant ADT in our model, which had been rarely examined in previous series. In this context, Togashi et al. did not report any influence on deterioration of postoperative LUTS, consistent with our findings^13^.

Our study has limitations. First, our data originate from a highly specialized single tertiary referral center and are not necessarily generalizable. Direct comparison to open radical prostatectomy is not possible since our center performs only RARP. However, the presented findings are highly consistent with previous series. Second, LUTS represent a patient-reported outcome. Our data was not complemented by ultrasound post void residual urine (PVR) or maximum urinary flow rates after 12 months to objectively measure the effect of RARP on micturition^28^. However, direct post-RARP PVR are institutionally measured by suprapubic catheter, which is only removed if PVR is repeatedly below 100 ml. Finally, for purpose of post-RARP erectile function recovery, several patients medicate with phosphodiesterase-5 inhibitors (PDE-5I), which are known to alleviate LUTS in patients with concomitant BPH-LUTS^21^. Despite removal of the prostate a remaining effect on smooth muscle relaxation in the bladder and supporting vasculature is possible, thus potentially further improving LUTS post-RARP^34^. However, in our cohort higher LUTS severity was associated with lower potency rates: i.e. for mild vs. severe LUTS the corresponding preoperative potency rates were 52 vs. 37%. Such distribution strengthens our post-RARP findings, i.e. greatest LUTS improvement rates in exactly those with severe preoperative LUTS.

## Conclusions

In one of the largest RARP cohorts with preoperative moderate or severe LUTS burden, we observed substantial proportions of patients with marked improvements of LUTS at 12 months after RARP, which translates to improvement of QoL. LRM revealed greatest benefit in those with preoperatively greatest LUTS burden, prostate enlargement, lower BMI, younger age and the event of urinary continence recovery. In presence of such findings, such postoperative functional and QoL prospects are integral for patient counseling regarding to the prostate cancer treatment.

## Supplementary Information

Supplementary Information.

## Abbreviations

ADT: Androgen deprivation therapy, i.e. antiandrogen and/or luteinizing hormone-releasing hormone antagonist or agonist therapy
BMI: Body mass index
BPH: Benign prostate hyperplasia
CAPRA-S: Postsurgical Cancer of the Prostate Risk Assessment Score
IPSS: International Prostate Symptoms Score
LRM: Logistic regression model
LUTS: Lower urinary tract symptoms
MCID: Marked clinically important differences
MVA: Multivariable cox regression analyses
PCa: Prostate cancer
PSA: Prostate specific antigen
QoL: Quality of life
RARP: Robot-assisted radical prostatectomy
NS: Nerve sparing
