# Supplementary material for: Improvement of quality of life and symptom burden after robot-assisted radical prostatectomy in patients with moderate to severe LUTS
Source: Sci Rep. 2021 Aug 18;11:16757. doi: 10.1038/s41598-021-95525-2 (PMC8373967; doi:10.1038/s41598-021-95525-2)
Supplement: Supplementary file 1 — Supplementary Information. [file 41598_2021_95525_MOESM1_ESM.pdf]

Supplemental Table 1: Multivariable linear regression of change of LUTS 12 months after robot-assisted radical prostatectomy

Value	Estimates[a]	95% CI	*p-value*
**Age, years, cont.**	0.01	-0.01 – 0.02	0.384
**BMI, kg/m2, cont.**	0.09	0.05 – 0.12	<0.001
**Preoperative moderate LUTS burden (vs. none/mild[REF.])**	-6.23	-6.47 – -6.00	<0.001
**Preoperative severe LUTS burden (vs. none/mild[REF.])**	-15.3	-15.83 – -14.81	<0.001
**Prostate gland weight, cont. (per 10g)**	-0.29	-0.35 – -0.24	<0.001
**Urinary continence recovery within 12 months after RARP, cont.**	-1.94	-2.30 – -1.58	<0.001

[a] - adjusted for surgical experience, preoperative PSA, preoperative medication with alpha-blockers, 5-alpha-reductase inhibitors, neoadjuvant androgen deprivation, CAPRA-S score, nerve-sparing status and bladder neck reconstruction width

CAPRA-S - the postsurgical Cancer of the Prostate Risk Assessment score, IPSS - International Prostate Symptom Score, LUTS - lower urinary tract symptoms, RARP - robot-assisted radical prostatectomy
